# Characteristics and Mechanisms of Cardiopulmonary Injury Caused by Mine Blasts in Shoals: A Randomized Controlled Study in a Rabbit Model

**DOI:** 10.1371/journal.pone.0081310

**Published:** 2013-12-16

**Authors:** Gengfen Han, Ziming Wang, Jianmin Wang, Weixiao Yang, Jing Chen, Jianyi Kang, Sen Zhang, Aimin Wang, Xinan Lai

**Affiliations:** 1 Department of Orthopedics, Daping Hospital, Third Military Medical University, Chongqing, China; 2 Research Institute of Surgery, Daping Hospital, Third Military Medical University, Chongqing, China; 3 Department of Ultrasound, Daping Hospital, Third Military Medical University, Chongqing, China; University of São Paulo, Brazil

## Abstract

**Background:**

Because the characteristics of blast waves in water are different from those in air and because kinetic energy is liberated by a pressure wave at the water-air interface, thoracic injuries from mine blasts in shoals may be serious. The aim of the present study was to investigate the characteristics and mechanisms of cardiopulmonary injury caused by mine blasts in shoals.

**Methods:**

To study the characteristics of cardiopulmonary injury, 56 animals were divided randomly into three experimental groups (12 animals in the sham group, 22 animals in the land group and 22 animals in the shoal group). To examine the biomechanics of injury, 20 animals were divided randomly into the land group and the shoal group. In the experimental model, the water surface was at the level of the rabbit's xiphoid process, and paper electric detonators (600 mg RDX) were used to simulate mines. Electrocardiography and echocardiography were conducted, and arterial blood gases, serum levels of cardiac troponin I and creatine kinase-MB and other physiologic parameters were measured over a 12-hour period after detonation. Pressures in the thorax and abdomen and the acceleration of the thorax were measured.

**Conclusion:**

The results indicate that severe cardiopulmonary injury and dysfunction occur following exposure to mine blasts in shoals. Therefore, the mechanisms of cardiopulmonary injury may result from shear waves that produce strain at the water-air interface. Another mechanism of injury includes the propagation of the shock wave from the planta to the thorax, which causes a much higher peak overpressure in the abdomen than in the thorax; as a result, the abdominal organs and diaphragm are thrust into the thorax, damaging the lungs and heart.

## Introduction

Amphibious warfare is one of the most important naval battle forms. In the beach landing stage, landing personnel wade shoreward from a landing ship or boat. Typically, the opposing personnel place a large number of mines in shoals, hindering the landing and causing many casualties [1], [2]. In the landings at the battle of Normandy, the Germans laid approximately 6,500,000 mines in the coastline, which led Allied forces to suffer heavy casualties [3]. Moreover, mines remain in very shallow water, surf zones and on the beach. The Navy and Marine Corps have spent nearly a decade of research and at least $70 million on engineering and testing, but they are far from having suitable equipment to detect and breach minefields [4], which may result in civilian casualties.

The high-pressure wave of an underwater blast encompasses a larger area than a blast in air [5]. Underwater blast waves are characterized by a high peak pressure and a short positive action duration. Moreover, water is 800-fold denser than air and is non-compressible [6]. Thus, blast waves in water propagate rapidly, with a slow rate of dissipation, and have a greater potential for injury than an air explosion [7]. Due to the liberation of kinetic energy by the pressure wave at the surface, at which the two different media join [8], internal organs present at the gas-liquid interface can be severely damaged, particularly the lung, which is a gas-containing viscus. Of special significance in the pathogenesis of mine-blast wounds in shoals is pneumonia followed by arterial air embolism and encephalopathy [9]. However, the characteristics and mechanisms of thoracic injuries after mine blasts in shoals remain unclear. Elucidating this problem may help to determine the proper treatment and protective measures. The aim of the present study was to investigate the characteristics and mechanisms of cardiopulmonary injury caused by mine blasts in shoals.

## Materials and Methods

### Subjects and Groups

Seventy-six healthy, male, New Zealand rabbits (supplied by Flied Surgery Institute, Third Military Medical University, Chongqing, China) were housed in 12 h light-dark conditions with free access to water and standard laboratory chow. Animal procedures were performed strictly in accordance with the guidelines for the use of laboratory animals approved by the Ethics Committee of the Third Military Medical University, Chongqing, China.

To study the characteristics of cardiopulmonary injury, 56 animals were divided randomly into three experimental groups (12 animals in the sham group, 22 animals in the land group and 22 animals in the shoal group) in accordance with a random number table. To examine the biomechanics of injury, 20 animals were divided randomly into two experimental groups (10 animals in the land group and 10 animals in the shoal group) in accordance with a random number table.

### Animal Preparation

The animals were anesthetized by the intravenous injection of pentobarbital (30 mg/kg) into the marginal vein of the ear. Trauma was not induced until the rabbit was in deep anesthesia. Additional doses of 10 mg/kg were administered hourly to maintain anesthesia. Local anesthesia was administered with lidocaine hydrochloride before femoral artery cannulation. All animals received a intravenous injection of 2 mg/kg carprofen after cannulation to prevent pain. The left femoral artery was dissected and cannulated with a three-way tube used to collect blood samples and measure mean arterial pressure (MAP) at baseline (after anesthetization but not fixed to the metal holder) and at 3, 6 and 12 hours after impact using an ECG monitor (Philips VM8).

The experiment was implemented in the center of a pool. The forelimbs of the anesthetized animals were fixed to a special metal holder, and the height of the metal holder was adjusted to ensure that the double hindpaws stood on the ground. The fore-and-aft clearance of the bilateral hindpaws was 11 cm, and the space between the left and right hindpaws was 9 cm. To simulate the damaging effects of anti-personnel mines, rabbits weighing 2.19±0.12 kg (range of 2.00–2.40 kg) were used. RDX paper electric detonators (600 mg, 845 Factory, Chongqing, China) were used to simulate mines and were placed under the rabbit's right foot and ignited electronically. Pilot studies showed that the magnitude of the blast was scaled to the size of the animal. The wounds of all rabbits were sutured and bandaged immediately after the detonation. In the shoal group, the water reached the rabbit's xiphoid process. The water was maintained at 20±2°C, and the room temperature was maintained at 22±2°C. In the land group, the water in the pool was extracted, and the ground was covered with sandy soil. The sham group simulated the shoal group without detonation.

The animals were immobilized on the operating table after anesthetization. After immobilization, a 20% sodium sulfide solution was used to remove hair from the limbs of the rabbits. Three standard limb leads were recorded via four alligator clip electrodes on the forelimbs and hindlimbs with an electrocardiograph (CardiMAX FX-7202), a vertical calibration of 10 mm/mV and a horizontal paper speed of 25 mm/second. Printouts were made at baseline and every 30 min after detonation or as necessary. ST segment depression was defined as measurements from lead II indicating that the ST segment fell more than 0.05 mv. A Q wave greater than 1/4 of the QRS wave was defined as a pathological Q wave.

Echocardiography (Philips iU22) was used to measure the left ventricular end diastolic dimension (LVIDd), left ventricular end systolic dimension (LVIDs), heart rate, stroke volume (SV), fractional shortening, ejection fraction and cardiac output (CO) and to diagnose mitral regurgitation at baseline and at 3, 6 and 12 hours after impact. All data and images were obtained with the same probe (Philips S5-1). All of the rabbits underwent spiral CT at 6 hours after impact, and injuries were diagnosed and analyzed on coronal, axial and reconstructed images.

### Sample Preparation

Blood samples (2.5 ml) were acquired at baseline and 3, 6 and 12 hours after detonation. A portion of each sample (2 ml) was centrifuged at 1,500× g for 10 minutes to obtain serum, which was stored at −70°C for analysis of the cardiac troponin I (cTnI) and creatine kinase-MB (CK-MB) levels. The levels of these two specific proteins in the serum were determined using an enzyme-linked immunosorbent assay kit (Westang, Shanghai, China). The OD value was measured at 450 nm (Multiskan MK3, Thermo Labsystems, Shanghai, China). The remaining 0.5 ml was used for analysis of the arterial partial pressure of oxygen (PO_2_), partial pressure of carbon dioxide (PCO_2_), pH, Na^+^, K^+^, Ca^2+^, lactate levels, hemoglobin and blood glucose.

### Pathological Examination

After the 12-hour observation period, the animals were sacrificed via an overdose of pentobarbital until the ECG became isoelectric. The lungs were removed and photographed. Computer-aided Design software 2007 (CAD 2007) was used to measure the area of pulmonary hemorrhage. Samples (1 cm long) were cut in a downward direction from the left ventricular apex and right lower lung by the same person to ensure that all samples were taken from the same spot. The samples were immediately soaked in 4% formaldehyde at 4°C and then sectioned into 5 µm sections for morphologic evaluation. Slides were stained with hematoxylin and eosin (Chemical Reagent Factory) and were examined under a light microscope (IX 50, Olympus, Tokyo, Japan). Lung edema formation, infiltration of inflammatory cells and pulmonary architecture were assessed in a blinded manner. Lung injury was scored categorically according to Gu's protocol [10]: Grade 0, normal appearance; Grade 1, mild moderate interstitial congestion and neutrophil leukocyte infiltrations; Grade 2, perivascular edema formation, partial destruction of pulmonary architecture and moderate neutrophil leukocyte infiltration; and Grade 3, complete destruction of the pulmonary architecture and dense neutrophil leukocyte. For each section, five random areas were selected, and the average score was recorded. The liver, spleen, kidneys, spinal cord and cerebral cortex were also examined.

### Biomechanics Recording System

Two pressure transducers (2 mm diameter, 4.5 mm length, 0.2 g Mass, 2 MPa capacity) were used for the peak overpressure tests. One pressure transducer (1357, Decheng, Xian, China) was located in the abdomen, and the transducer was fastened to the muscle of the abdominal wall at the medioventral line 25 cm to the detonator. The other transducer (1353, Decheng, Xian, China) was inserted into the thorax through the esophagus by the following procedures. A neck incision was made to expose the esophagus, after which the esophagus was dissected, and the pressure transducer was inserted. The position of the transducer was adjusted under a digital X-ray machine to ensure that the transducer was located at the level of T5. The pressure transducer was ligated to the esophagus and the surrounding muscles. The differential pressure between the thorax and abdomen was calculated according to the following formula: Differential pressure between the thorax and abdomen = Peak overpressure in the thorax - Peak overpressure in the abdomen.

A uni-axial accelerometer (A051068, Measurement Specialties, Hampton, VA) was used for the peak positive acceleration and peak negative acceleration tests. Four pieces of sheet metal, each with a hole in the center, were welded around the accelerometer. The accelerometer was fastened to the sixth and seventh ribs using copper wires run through the sheet metal pieces, ensuring that the copper wires did not penetrate the pleura. The sensitive axis of the accelerometer was oriented toward the thoracic cavity. Signals were acquired using a TST6150 (Chengdutest, Chengdu, China) and were analyzed with DAP6.01. The sampling rate of the data acquisition system was 200 kHz. First, the signal frequency spectra were analyzed by using the fast Fourier-transformed linear spectrum. Second, the infinite impulse response filter was used, and the cut-off frequencies were set at 100 kHz.

### Statistical Analysis

Data are presented as the mean ± standard error. Two factors with a four-level mixed repeated measures ANOVA were used to explore significant differences between and within groups, and the statistical analysis was performed using SPSS16.0. The dependent variables were the respiratory and circulatory parameters and the CK-MB, cTnI and lactate levels. Two-tailed independent sample t-tests were used to examine differences in the area of pulmonary hemorrhage and the biomechanical variables between the shoal group and the land group. Histological lung injury scoring was expressed as a box-and-whisker plot, and an analysis of variance that used the Kruskal-Wallis nonparametric test was then performed for comparison. Pearson chi-square tests were performed for categorical data, except when the cell counts did not exceed 5 specimens or the total number of cases was less than 40. When this event occurred, Fisher's exact tests were used. Means are depicted inside 95% confidence intervals, and the alpha value was set at 0.05. Post-hoc tests were made for both the main effects and the interaction effects using the least squares difference (LSD) method.

## Results

### Animal Experiment and Postmortem Examination

Two animals died in the shoal group; one died 4 hours after exposure due to severe lung damage, and the other died 10 hours after exposure due to ventricular fibrillation. No animals died in the land group.

**Figure 1:**
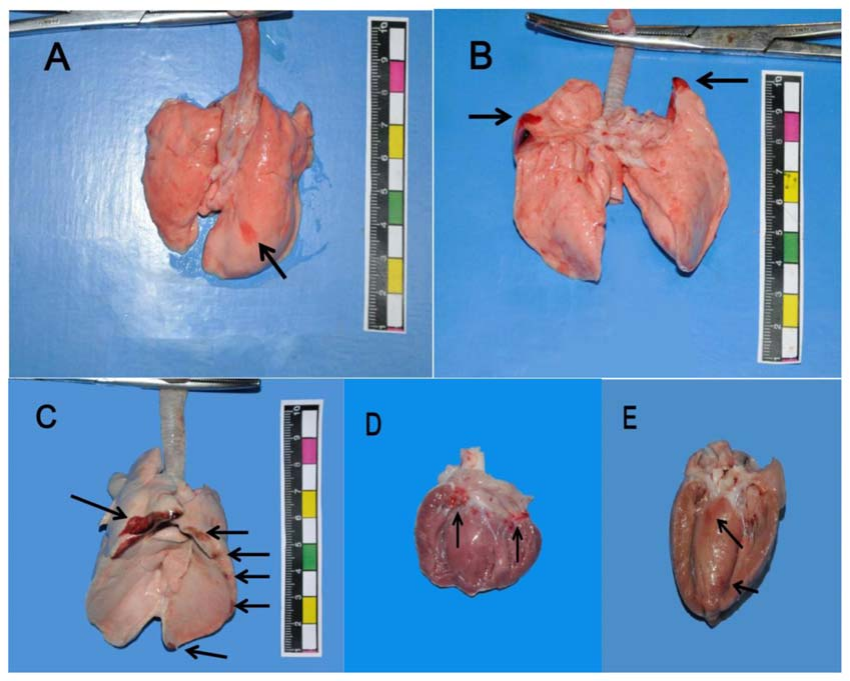
The heart and lung contusions (arrows) generated by the mine blast. Hemorrhage on the surface of the lung lobes in the land group (A). Hemorrhage at the apex of the lungs (B), at the lung tissue around the heart and above the diaphragm in the shoal group (C). Mitral valve hemorrhage (D) and myocardial contusion (E) in the shoal group.

Necropsy revealed scattered petechiation and hemorrhaging on the surfaces of the lung lobes in seven animals from the land group (Fig. 1A). However, animals in the shoal group were observed to have extensive hemorrhaging at the lung boundaries, including the bilateral apex of the lungs, around the heart and above the diaphragm (Fig. 1B, C). The area of pulmonary hemorrhage in the shoal group was 2.42±0.42 cm^2^, whereas it was 0.53±0.18 cm^2^ in the land group, and the difference was significant (p<0.01). Thirteen animals exhibited mitral valve contusions in the shoal group (Fig. 1D) compared with 4 animals in the land group, the difference was significant (p<0.01). Three animals presented myocardial contusions in the shoal group (Fig. 1E) compared with zero in the land group, and the difference was significant (p<0.05).

### Pathology Results

**Figure 2:**
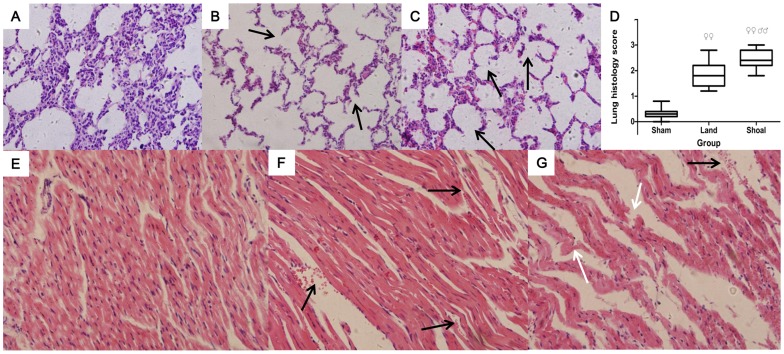
Photomicrographs of the lung and heart tissues (H&E, ×400). Normal pulmonary histology in the sham group (A). Alveolar rupture (black arrow) in the land group (B). Alveolar congestion and hemorrhage and breakdown of the alveolar architecture (black arrow) in the shoal group (C). Histopathological scoring data of lung injuries presented in a box-and-whisker plot (the boxes are constructed with 25% and 75% confidence intervals, median and maximum or minimum individual values) (D), ^♀♀^ p<0.01 versus the sham group, ^♂♂^ p<0.01 versus the land group. Normal heart histology in the sham group (E). Myocardial edema and congestion in the mesenchyme (black arrow) in the land group (F). Myocardial edema, hemorrhage (black arrow) and myofibrillar disarray and breakage (white arrow) in the shoal group (G).

Compared with the sham group (Fig. 2A), the lung morphology revealed alveolar hyperemia, an inflammatory reaction and destruction of the alveolar architecture (black arrow) in the land group (Fig. 2B). In the shoal group, histopathological observation showed obvious hyperemia, edema and marked destruction of the alveolar architecture (black arrow), in addition to cell infiltration and loss of alveolar space (Fig. 2C). These changes are demonstrated in the box-and-whisker plot of the scoring data (Fig. 2D).

The morphology and structure of the myocardium were normal in the sham group (Fig. 2E). The morphological structure of the myocardium changed in the land group, including swelling of cardiac myocytes and congestion in the mesenchyme (black arrow) (Fig. 2F). However, the heart injuries were more serious in the shoal group than in the land group. The pathological changes of the myocardial tissue included edema and hemorrhage (black arrow), myofibrillar disarray and rupture of the muscle fibers (white arrow) (Fig. 2G).

### Electrocardiogram

**Figure 3:**
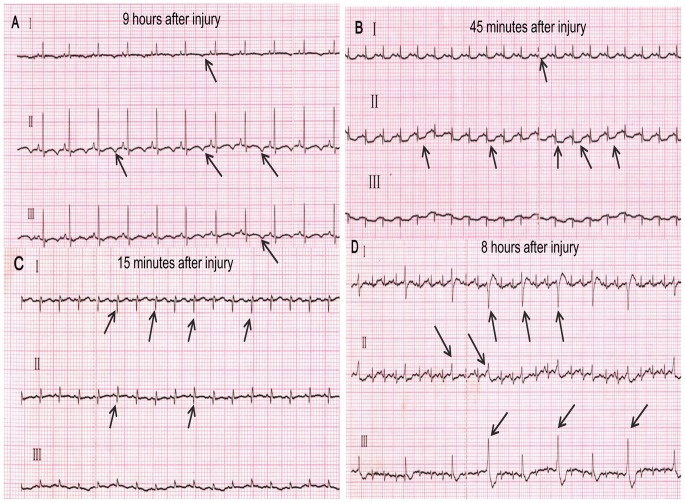
Electrocardiographic changes following blast injury. A peaked T-wave (black arrow) was observed at leads I, II and III in this animal from the land group at 9 hours after injury (A). ST-segment reduction (black arrow) was observed after 45 minutes at leads I and II in this animal from the shoal group (B). A pathological Q wave (black arrow) was observed at leads I and II in this animal from the shoal group at 15 minutes after injury (C). Frequent premature ventricular contraction waves (black arrow) were observed at leads I, II and III in this animal from the shoal group at 8 hours after injury, and this abnormality lasted for approximately 1 hour before spontaneously reverting to sinus rhythm (D).

Animals in the sham group did not exhibit any abnormalities. Except for one case of T-wave abnormalities (Fig. 3A), there were no abnormalities in the land group. By contrast, in the shoal group, ST-segment reduction was observed in 5 animals (Fig. 3B), and pathological Q waves (Fig. 3C) were observed in 6 animals; the incidence differed significantly compared with the land group (p<0.05). Frequent premature ventricular contractions (Fig. 3D) were observed in 2 animals, and T-wave abnormalities were observed in 1 animal, but the differences between groups were not significant.

### Echocardiogram

**Figure 4:**
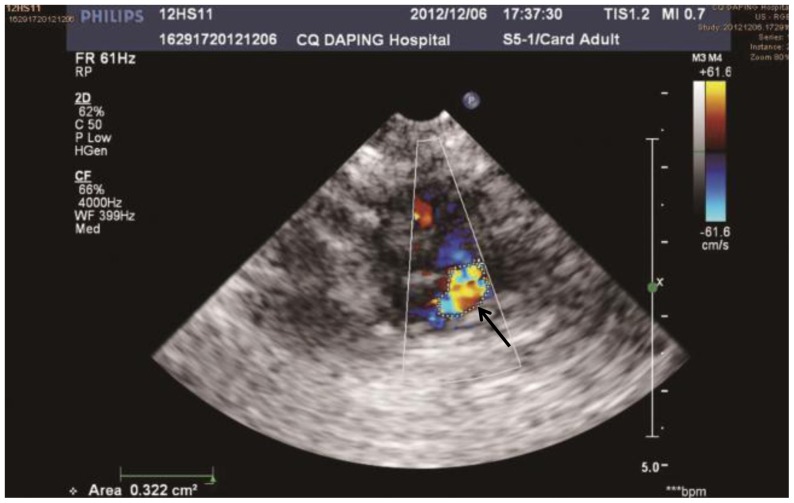
The morphological characteristics of mitral regurgitation. The area bounded by the dotted lines (black arrow) of regurgitation was measured in the animals in the shoal group.

No mitral regurgitation was observed in the sham group or the land group, but the echocardiogram revealed mitral regurgitation in 9 animals in the shoal group; the difference was significant compared with the land group (p<0.05). The degree of mitral regurgitation was determined by the area of the regurgitant flow (Fig. 4) at the level of the valve. The average area of the regurgitant flow was 0.37±0.06 cm^2^ at 12 hours after impact. The LVIDd, LVIDs, heart rate, fractional shortening and ejection fraction did not show any significant differences.

### Circulatory Effects

**Figure 5:**
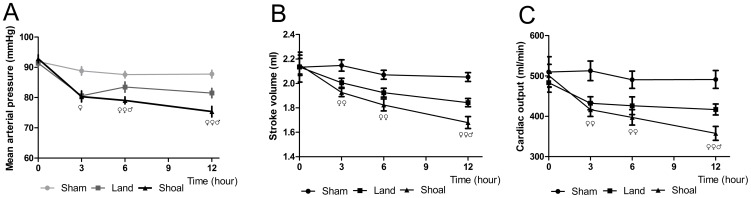
The circulatory parameters in the rabbits. Mean arterial pressure (A), stroke volume (B) and cardiac output (C) in the shoal group compared with the sham group and the land group. ^♀^ p<0.05, ^♀♀^ p<0.01 versus the sham group and ^♂^ p<0.05, ^♂♂^ p<0.01 versus the land group.

In the shoal group, the MAP declined at 3 (80.31±1.74 mmHg, p<0.01), 6 (79.06±1.16 mmHg, p<0.01) and 12 hours (75.38±1.74 mmHg, p<0.01) after impact compared with baseline (92.83±1.32 mmHg). The MAP was significantly decreased at 3 hours (p<0.05), 6 hours (p<0.01) and 12 hours (p<0.01) in the shoal group compared with the sham group and at 6 hours (p<0.05) and 12 hours (p = 0.01) compared with the land group (Fig. 5A).

In the shoal group, the SV was markedly declined at 3 (1.93±0.04 ml, p<0.05), 6 (1.82±0.04 ml, p<0.01) and 12 hours (1.68±0.04 ml, p<0.01) after impact compared with baseline (2.15±0.09 ml), and the CO was markedly lower at 3 (416.38±18.30 ml/min, P<0.05), 6 (397.32±20.58 ml/min, p<0.05) and 12 hours (357.80±16.85 ml/min, p = 0.001) compared with baseline (500.79±28.47 ml/min).

The SV in the shoal group was significantly decreased compared with the sham group from 3 to 12 hours after impact (p<0.05) (Fig. 5B). The difference in SV between the shoal group and the land group was significant at 12 hours (p = 0.05). The CO was declined between 3 and 12 hours after impact in the shoal group compared with the sham group (p<0.05) (Fig. 5C). Compared with the land group, the CO in the shoal group demonstrated a more marked decrease at 12 hours (p<0.05). There were no significant overall differences in SV or CO between the land and shoal groups, but there was a significant difference between the sham and shoal groups (p<0.01).

### Respiratory Effects

**Figure 6:**
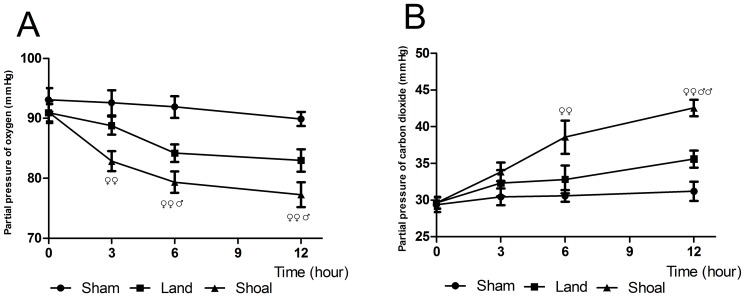
The respiratory parameters in the rabbits. The partial pressure of oxygen (A) and partial pressure of carbon dioxide (B) in the shoal group compared with the sham group and the land group. ^♀^ p<0.05, ^♀♀^ p<0.01 versus the sham group and ^♂^ p<0.05, ^♂♂^ p<0.01 versus the land group.

In the shoal group, the PO_2_ was markedly declined at 3 (82.85±1.69 mmHg, p<0.01), 6 (79.35±1.64 mmHg, P<0.01) and 12 hours (77.25±1.80 mmHg, P<0.01) after impact compared with baseline (91.00±1.82 mmHg). The PO_2_ was decreased at 3 (p<0.05), 6 (p<0.05) and 12 hours (p<0.01) in the shoal group compared with the sham group and was decreased at 6 hours (p<0.05) and 12 hours (p<0.05) compared with the land group (Fig. 6A). The PO_2_ showed significant overall differences between the land group and the shoal group (P<0.01).

The PCO_2_ in the shoal group was markedly increased at 3 (33.85±1.27 mmHg, p<0.01), 6 (38.60±2.27 mmHg, p<0.01) and 12 hours (42.55±1.13 mmHg, p<0.01) after impact compared with baseline (29.65±0.72 mmHg). The PCO_2_ was significantly increased at 6 hours (p<0.01) and 12 hours (p<0.01) in the shoal group compared with the sham group and was also significantly increased at 12 hours compared with the land group (P<0.01) (Fig. 6B). The PCO_2_ showed significant overall differences between the land group and the shoal group (P<0.01).

### Alterations of Serum CK-MB and cTnI Levels

**Figure 7:**
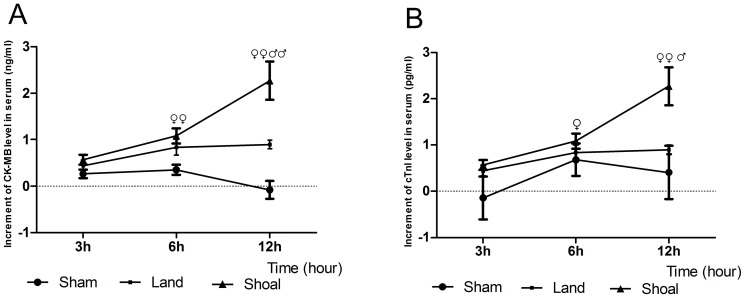
Alterations of serum CK-MB and cTnI levels. Increments of CK-MB (A) and cTnI (B) levels in the serum at different time points. ^♀^ p<0.05, ^♀♀^ p<0.01 versus the sham group and ^♂^ p<0.05, ^♂♂^ p<0.01 versus the land group.

The level of CK-MB in the shoal group was markedly increased at 6 hours (1.08±0.15 ng/ml, p<0.01) and 12 hours (2.27±0.27 ng/ml, p<0.01) compared with the sham group (Fig. 7A). The difference between the shoal group and the land group was not significant at 3 hours or 6 hours, but it was significant at 12 hours (p<0.01).

The cTnI level in the shoal group was markedly increased at 6 hours (2.55±0.62 pg/ml, p<0.05) and 12 hours (5.67±1.02 pg/ml, p<0.01) compared with the sham group (Fig. 7B). The cTnI level in the shoal group was also significantly increased compared with the land group at 12 hours (p<0.05). The overall differences in the levels of these two markers were significant between the land group and the shoal group (CK-MB, p<0.01; cTnI, p<0.05).

### Blood Analysis

The lactate level was markedly increased at 6 hours (8.12±0.57 mmol/L, p<0.01) and 12 hours (10.00±0.85 mmol/L, p<0.01) after impact in the shoal group compared with baseline (4.46±0.64 mmol/L). The blood levels of lactate significantly differed between the shoal group and the land group at 3 (p<0.05), 6 (p<0.01) and 12 hours (p<0.05) (Table 1). pH, Na^+^, K^+^, Ca^2+^, hemoglobin and blood glucose did not show any significant differences.

**Table 1:** Lactate production in rabbits at different time points.

	Baseline	3 h	6 h	12 h
Sham	3.88±0.41	3.48±0.45	3.46±0.50	3.64±0.50
Land	4.17±0.43	3.84±0.31	5.13±0.74	7.18±1.04
Shoal	4.46±0.64	5.02±0.36♀♀ ♂	8.12±0.57♀♀ ♂♂	10.00±0.85♀♀ ♂

♀♀ p<0.01 versus the sham group and.

♂ p<0.05,

♂♂ p<0.01 versus the land group.

### Imaging Examination

**Figure 8:**
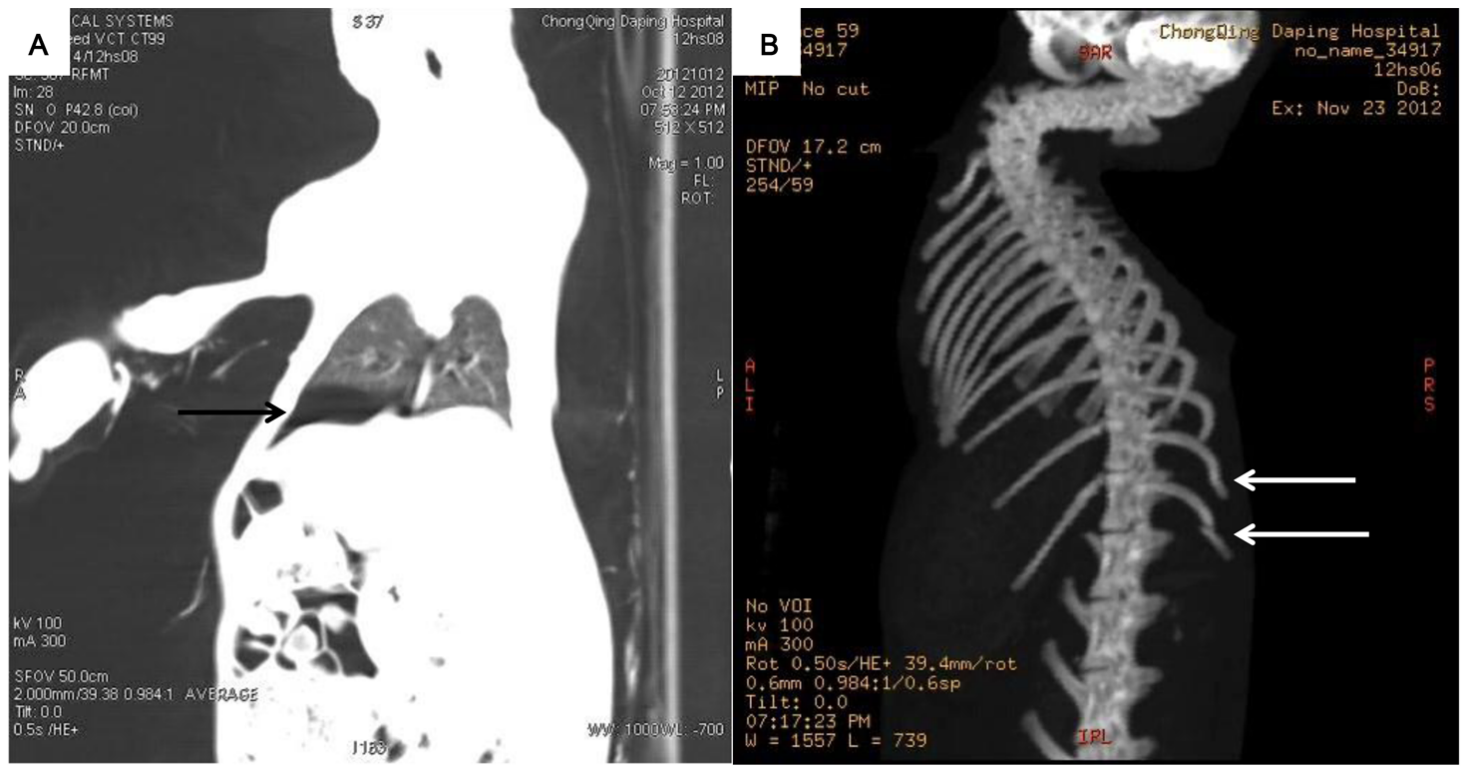
The imaging results after the blast injury. Pneumothorax (A, black arrow) and rib fractures (B, white arrows) were identified by CT in the shoal group at 6 hours after the impact.

CT revealed pneumothorax in 4 animals (Fig. 8A) and rib fractures in 3 animals (Fig. 8B). However, in the land group, no cases of pneumothorax or rib fracture were observed. The difference between the two groups in the incidence of pneumothorax was statistically significant (p<0.05).

### Biomechanics Recordings

The peak overpressure recorded in the thorax was 118.19±4.07 kPa in the shoal group and 22.97±1.02 kPa in the land group, and the difference was significant (p<0.01). The peak overpressure recorded in the abdomen was 454.06±13.72 kPa in the shoal group, which was significantly different from that in the land group (53.61±3.31 kPa, p<0.01). The differential pressure between the thorax and abdomen was 335.88±14.55 kPa in the shoal group, which was significantly different from the 30.64±3.81 kPa recorded in the land group (p<0.01).

The peak positive acceleration of the thorax was 12.39±0.83 g in the shoal group, which significantly differed from that in the land group (3.58±0.30 g, p<0.01). The peak negative acceleration of the thorax was 8.64±0.57 g in the shoal group, and 2.63±0.21 g in the land group, and the difference was significant (p<0.01).

## Discussion

Mine blasts in shoals can injure distant vital organs, including the lungs and heart due to the high pressure wave created in the water. The aim of the present study was to investigate the characteristics and mechanisms of cardiopulmonary injury caused by mine blasts in shoals. Not only the macroscopic and microscopic observations but also the ECG and markers of myocardial injury demonstrated that the injuries in the shoal group were more serious than those observed in the land group. The respiratory and circulatory parameters further showed that the lungs and heart were more seriously damaged in the shoal group than in the land group. These characteristics were in line with the peak overpressure and peak acceleration recorded in the thorax, which were significantly higher in the shoal group than in the land group. The rabbit model was chosen because the alveolar diameter and surface area in relation to oxygen consumption and metabolism are proportional to that of humans [11]. This scaling can allow accurate comparisons of physiological injuries between rabbits and humans. The magnitude of the detonator used in the study was also scaled to the size of the rabbits.

Determining the mechanisms leading to thoracic visceral damage resulting from mine blasts in shoals is important for developing protective equipment. Cooper et al [12] have suggested that the direct transmission of stress waves plays a dominant role in lung parenchymal injury from blast loading and that gross thoracic compression is not the primary injury mechanism in air blasts. By contrast, Coppel et al [13] have suggested that blast injuries to the lungs are due to direct compression. In the present study, scattered petechiation and hemorrhaging were observed on the surfaces of the lung lobes in animals from the land group. However, in the shoal group, hemorrhaging at the lung boundaries was observed, particularly around the heart, above the diaphragm and at the bilateral apex of the lungs. It appears that the high peak overpressure of the blast wave was transmitted from the bottom up and that the intra-abdominal pressure increase then thrust the abdominal organs and diaphragm into the thorax, damaging the lungs. This injury mechanism is similar to the hypothesis of Dancewicz et al, who suggested that the lung injuries caused by explosions were due to the lung deformation caused by the sudden pressure increase in the thoracic cavity and the subsequent volume reduction in the thorax [14]. The results of the biomechanical analysis also demonstrate this viewpoint as the differential pressure between the thorax and abdomen in the shoal group was notably higher than that in the land group. Another possible explanation for the injuries is that water is denser than air, and therefore, the shear waves produced strain at the water-air interface. The biomechanical test results showed that the peak positive and negative acceleration values in the thorax were significantly higher in the shoal group than in the land group. Therefore, shear waves may produce strain, leading to strong vibrations of the thorax and viscera that cause injury. This may be the source of lung hemorrhaging observed at the boundary around the heart.

In the present study, the most prominent disturbances in the physiological parameters were the changes in respiration and circulation. The PO_2_ was markedly decreased and the PCO_2_ was significantly elevated at 12 hours compared with the land group after impact, indicating serious lung dysfunction in the shoal group. The PO_2_ decreased due to the primary lung contusions, but other causes were also present, including hemorrhage occluded alveoli, ventilation-perfusion mismatch caused by pulmonary hemorrhage [15] and the release of inflammatory mediators leading to diminished respiratory function [16]–[19]. After a marked decrease, the MAP in the land group recovered to a level close to that of the sham control at 6 hours after impact, whereas the MAP continued to fall after 3 hours in the shoal group. Although there were no significant changes in heart rate, the SV and CO gradually declined, resulting in a more significant decrease in the MAP of the shoal group than in that of the land group at 6 hours and 12 hours. The SV decline may have resulted from mitral regurgitation [20]. In addition, the SV may have declined as a result of massive pulmonary hemorrhage from disruption of the alveolar architecture and the formation of alveolar-venous fistulas resulting in air embolism [21]–[23], myocardial ischemia from hypoxia and air embolism in the coronary circulation [15]. Respiratory dysfunction, together with the SV decline, led to inadequate oxygen delivery, increasing the blood lactate concentration, which demonstrated peripheral anaerobic metabolism.

In the present study, CT scans showed that 20% of the animals in the shoal group had pneumothorax. Madill et al [24] have suggested that thoracic ultrasonography is a portable imaging tool that can be used to detect pneumothorax during an air evacuation, particularly tension pneumothorax, which is highly lethal [25]. If the diagnosis of pneumothorax is confirmed, proper management should be implemented immediately, such as thoracostomy [26], ventilation [27] or the insertion of an intercostal tube.

The ECG changes were one of the most interesting findings in this study. Rapid ECG changes, including pathological Q waves, ST-segment reduction and arrhythmia were demonstrated in the shoal group animals. Huller et al [28] found that underwater blasts also produced ECG changes indicative of myocardial injury, including ST-segment depression, Q waves and flat T-waves. Similarly, Carlsten et al [29] found that ST- segment depression and T-waves were common after detonation. These disturbances may be exacerbated and should be considered during evacuation and treatment.

In this study, the serum levels of CK-MB and cTnI were significantly increased in the shoal group compared with those in the land group at 12 hours after impact, which was in line with microstructural changes, indicating severe myocardial injury due to the mine blasts in shoals.

One limitation of the present study was that it included only one water depth, which did not represent the various damaging effects and mechanisms of injury. Additionally, a wounding mechanism that inflicted a traumatic injury was used in the study, and this complicating factor should have been avoided.

## Conclusions

Our study has demonstrated that severe cardiopulmonary injury and dysfunction result from mine blasts in shoals. Particularly noteworthy were the observed arrhythmia and pneumothorax, for which diagnostic and treatment delays may be lethal. These consequences must be considered when evacuating wounded individuals and diagnosing and treating injuries.

The mechanisms of cardiopulmonary injury may result from the greater density of water compared with air, causing shear waves that produce strain at the water-air interface. Another mechanism of injury includes the propagation of the shock wave from the planta to the thorax, which causes the peak overpressure to be much higher in the abdomen than in the thorax; as a result, the abdominal organs and diaphragm are thrust into the thorax, damaging the lungs and heart. These mechanisms may be important for the development of protective equipment.
